# Collagenous colitis presenting as spontaneous perforation in an 80 year old woman: Report of a Case

**DOI:** 10.1186/s12876-016-0533-1

**Published:** 2016-10-06

**Authors:** Andrew Mitchell, Alexandre Dugas

**Affiliations:** 1 Department of Anatomic Pathology and Cytology, Maisonneuve-Rosemont Hospital, 5415 Boulevard de L’Assomption, Montreal, Quebec H1T 2X1 Canada; 2 Department of Radiology, Maisonneuve-Rosemont Hospital, 5415 Boulevard de L’Assomption, Montreal, Quebec H1T 2X1 Canada

**Keywords:** Collagenous, Colitis, Perforation, Spontaneous, Case report

## Abstract

**Background:**

Perforation of the colon occurring during or shortly following colonoscopy or barium enema is a rare complication of collagenous colitis (CC). "Spontaneous" perforation in CC, in which no instigating factor is identified, is even less common, with only five cases reported to date. We report herein an additional case of spontaneous perforation in previously undiagnosed CC and review the clinical and pathological features of previously reported cases.

**Case presentation:**

An 80 year old woman presented to the emergency department with abdominal pain preceded by approximately one month of frequent non-bloody diarrhea. Abdominal CT showed parietal thickening of the colon at the splenic flexure with pneumatosis and signs of perforation. Segmental resection was performed. Pathologic examination showed the microscopic findings typical of CC complicated by several deep ulcers and perforation. One day following discharge from hospital abdominal pain and frequent non-bloody diarrhea recurred. The patient was managed conservatively and treated with oral budesonide with resulting resolution of symptoms.

**Conclusions:**

Spontaneous perforation is a rare and serious complication of CC. All patients to date have been female. In contrast to procedure-related perforation, which favors the right colon, spontaneous perforation in CC has in all cases involved the left colon. Knowledge of spontaneous perforation as a potential complication of previously undiagnosed CC may be helpful in the evaluation and management of patients presenting with colonic perforation, especially those with risk factors for CC.

## Background

The classical clinical and pathologic features of collagenous colitis (CC) are well known [1, 2]. Less appreciated is that perforation of the colon is a possible complication of CC [1, 2]. Perforation may be either procedure-related or "spontaneous", the former being more frequent and related to colonoscopy or barium enema [1, 3]. To date, only five examples of spontaneous perforation in CC have been reported [4–8]. We present herein a further case of spontaneous perforation complicating CC. A review of all cases of spontaneous perforation suggests a predilection for the left colon, distinct from the predominance of right-sided perforations in procedure-related cases.

## Case presentation

An 80 year old female came to the emergency department of our hospital complaining of abdominal pain of recent onset. She related a history of frequent episodes of non-bloody diarrhea of approximately one month's duration, unresponsive to loperamide. Her past medical history included surgical correction of rectal prolapse six months previously, implantation of a pacemaker, anticoagulation, osteoporosis, and hypothyroidism treated with levothyroxine.

Her temperature was normal. Physical examination revealed lower abdominal tenderness but was otherwise unremarkable. The white blood cell count was 8.1 ×10^9^/L (normal: 4.5-10.8 ×10^9^/L). The patient was admitted to hospital.

**Fig. 1:**
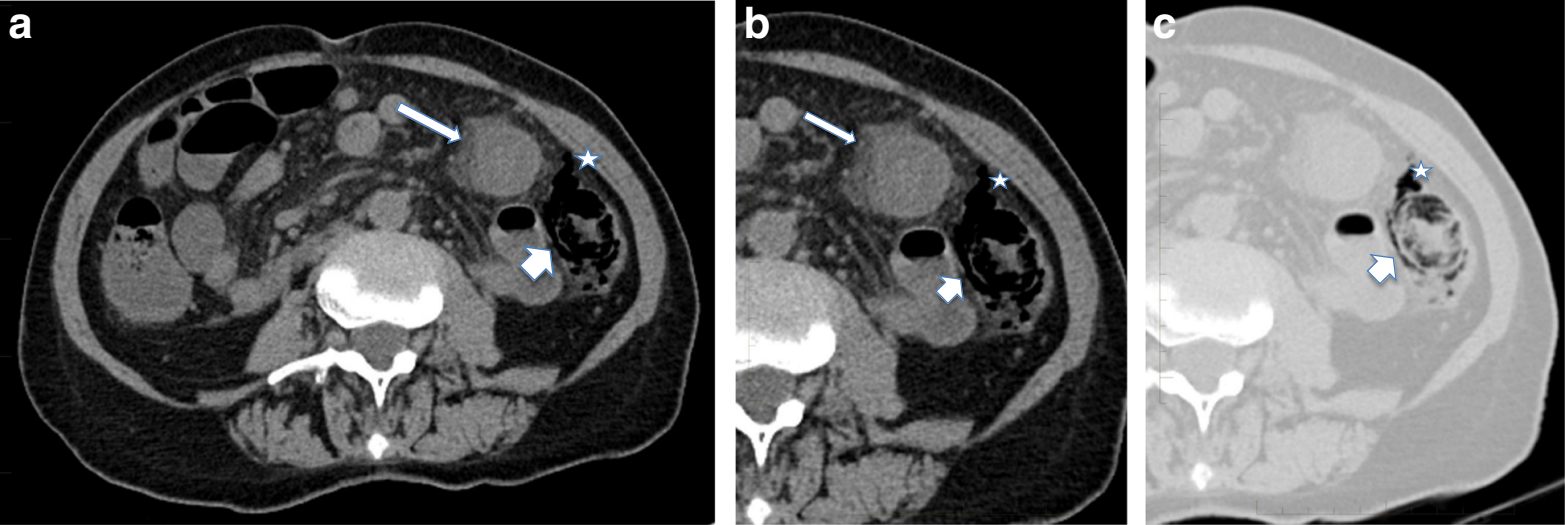
Radiologic images: **a** Axial view of unenhanced abdominal CT showing thickened bowel wall with fat stranding (*thin arrow*) involving the colonic splenic flexure. **b** and **c** Zoomed axial view of the left abdomen in soft tissue (**b**) and pulmonary (**c**) windows showing pneumatosis (*thick arrow*) and free air in the surrounding mesocolon (*star*)

Due to chronic renal failure unenhanced abdominal CT scanning was performed (Fig. 1). This showed a 15 cm length of colon at the splenic flexure with significant parietal thickening and fat stranding, as well as pneumatosis and free air in the surrounding mesocolon. Ischemic colitis was diagnosed based on location in a watershed area and signs of necrosis and perforation.

The following day the white blood cell count was 20.4 ×10^9^/L. In light of this and the CT scan findings, an exploratory laparotomy was performed which revealed a small amount of ascites. A 16 cm segment of thickened bowel from the splenic flexure was resected.

The postoperative course was uneventful. The patient was discharged on the eighth post-operative day. However, the next day she was readmitted to hospital with abdominal pain accompanied by at least ten episodes of non-bloody diarrhea occurring that day. An abdominal CT scan with rectal contrast showed no evidence of anastomotic leak or other abnormality. Stool culture and testing for Clostridium difficile toxin were negative. The patient improved with conservative management. Given the diagnosis of collagenous colitis (see pathologic findings below), treatment with oral budesonide was started and she was discharged on the eleventh day of her hospital stay. One month later she reported feeling well and having one to two stools of normal consistency per day.

### Pathologic findings

**Fig. 2:**
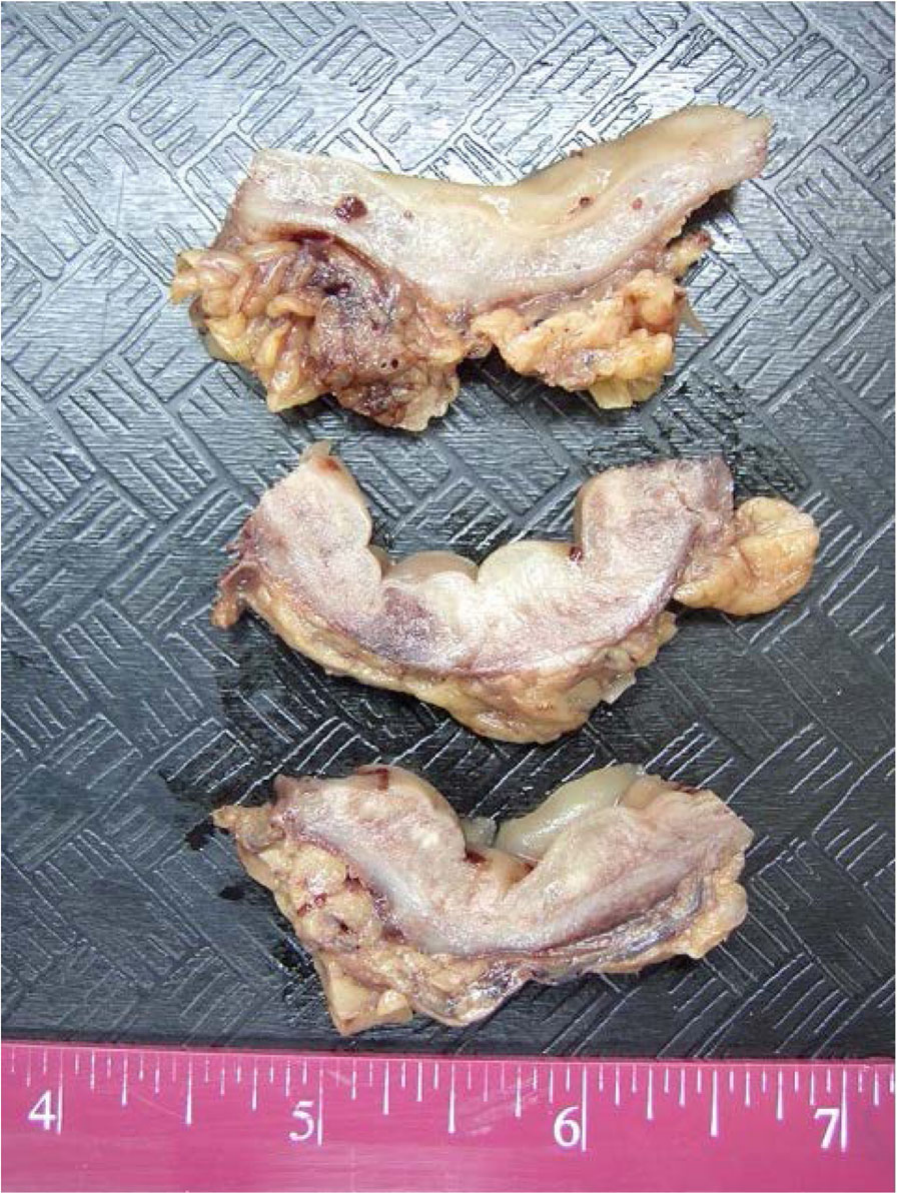
Macroscopy of the resected colon: Multiple cross sections of the bowel show normal appearing mucosa and a markedly thickened edematous wall measuring up to 15 mm

Macroscopic examination (Fig. 2) showed normal appearing mucosa but a markedly edematous and thickened wall measuring up to 1.5 cm.

**Fig. 3:**
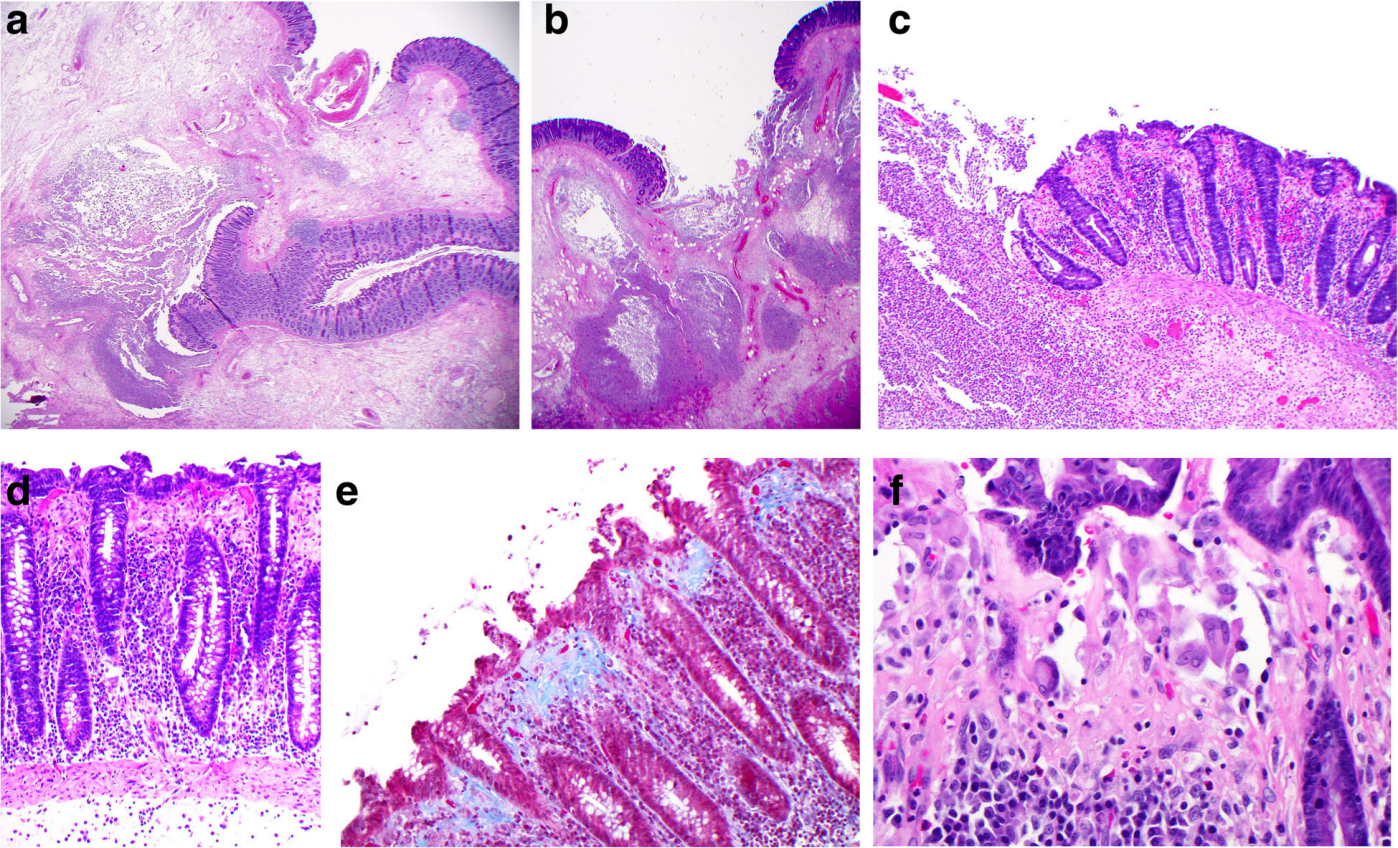
Microscopy of the resected colon: **a** and **b** Low power views (×12.5) of ulcerated bowel with abscess formation and transmural necrosis. Even at this low magnification the non-ulcerated mucosa shows clear evidence of collagenous colitis. **c** Medium power view (×100) of mucosal ulceration (*left*) and severe collagenous colitis (*right*). **d** High power view (×200) of the mucosa showing the features of collagenous colitis: a markedly thickened subepithelial collagen layer measuring up to 100 μm, surface epithelial flattening with loss of goblet cells, separation of the epithelium from the lamina propria, increased intraepithelial and lamina propria chronic inflammatory cells. **e** High power view (×200), Masson's trichrome stain, in which the thickened subepithelial collagen layer is stained blue. **f** Very high power view (×400) of multinucleated histiocytes

Microscopic examination (Fig. 3) showed the characteristic features of severe collagenous colitis throughout the segment of colon with areas of ulceration and abscess formation. The latter were transmural in at least two sites, compatible with perforation. Of note, subepithelial histiocytes, often multinucleated, were a prominent feature in the collagen band, but true multinucleated giant cells characteristic of the giant cell form of collagenous colitis [9] were not found. There was no evidence of acute ischemic colitis or inflammatory bowel disease.

Review of the prior rectal prolapse resection specimen confirmed the microscopic findings characteristic of prolapse. Histologic features of collagenous colitis were absent.

## Conclusions

Perforation of the colon in CC may occur in two settings: 1) procedure-related (during or shortly following colonoscopy or barium enema), and 2) "spontaneous", that is, unrelated to any intervention.

Although the first reported case of perforation was of the spontaneous type [4], subsequent reports have shown procedure-related perforations to be more frequent, with at least 22 reported cases [1, 10]. Patients with procedure-related perforations are mainly elderly women with a history of chronic diarrhea, with perforation occurring during the intervention or up to seven days afterwards [3, 5]. The endoscopist may note, presaging perforation, "a long, shallow, linear or serpiginous mucosal ulcer, appearing like a 'crack' or 'fracture' … usually orientated along the longitudinal axis of the colon and, in all cases, extended over several haustral segments" [11]. The right colon is the most common site, followed by the transverse colon, and, exceptionally, the left colon. As to pathogenesis, it is conjectured that the deposition of collagen in the lamina propria renders the mucosa stiff and non-distensible and therefore susceptible to "cracking" when, for example, air is introduced during colonoscopy, leading to perforation [1, 11].

Spontaneous perforation in CC has been described in five previous patients [4–8] (Table 1). In all cases except one (Freeman, [4]), photomicrographs are provided which demonstrate the characteristic histologic features of CC. All individuals have been female with a history of non-bloody diarrhea and no previous diagnosis of CC. Thus, colonic perforation was the initial manifestation of CC, with pathologic study of the resected bowel allowing diagnosis of the underlying disease. All patients recovered following resection of the perforated segment. Of note, all perforations occurred in the left colon, in contrast to the transverse/right-sided predominance in procedure-related perforations.

**Table 1:** Reported cases of spontaneous perforation in collagenous colitis

Case	Age/Sex	History of non-bloody diarrhea	Prior diagnosis of CC	Perforation site	Treatment	Outcome
Freeman [4]	37, F	3 weeks	No	Sigmoid	Laparotomy, no resection	Alive
Bohr [5]	56, F	At least 2 weeks	No	Splenic angle/descending colon	Segmental resection	Alive
Bennett [6]	67, F	2 months	No	Splenic flexure	Partial colectomy	Alive
Akamoto [7]	64, F	2 weeks	No	Descending colon	Left colectomy	Alive
Cottreau [8]	49, F	7 months	No	Descending colon	Left colectomy	Alive
Present case	80, F	1 month	No	Splenic angle	Segmental resection	Alive

*CC* collagenous colitis, *F* female

Regarding differential diagnosis, as the clinical findings of ischemic colitis (frequent) and CC with perforation (very rare) would seem to significantly overlap, diagnosis of CC will depend upon high clinical suspicion and histologic analysis of a biopsy or surgical resection specimen. Patients in both CC and IC tend to be middle-aged to elderly, with a female predominance in CC. IC classically presents with abdominal pain and blood in the stool, whereas CC is typified by frequent episodes of non-bloody diarrhea.

Risk factors for IC include a number of cardiovascular pathologies such as peripheral vascular disease, diabetes, dyslipidemia, heart failure and abdominal aortic aneurysm repair [12]. Risk factors for CC include autoimmune disease, malignancy, and organ transplantation [13]. Of the autoimmune diseases, celiac disease has the closest association (12.9-20 % of patients) [2, 13], with diabetes, thyroiditis, Sjogren's syndrome, spondylitis and pyoderma gangrenosum also described. Both IC and CC are associated with use of various medications [12, 13]. Digoxin and aspirin have been implicated in IC (and, of the illicit drugs, cocaine). Of the large number of medications associated with development of CC, noteworthy are proton pump inhibitors, beta-blockers, angiotensin II receptor antagonists, aspirin, NSAIDs and selective serotonin reuptake inhibitors.

Colonoscopy and histologic examination both provide findings that will usually allow separation of IC from CC. The colonoscopic appearance of the mucosa in CC is usually normal, whereas IC features a range of abnormalities including erosions, longitudinal ulcerations, petechial hemorrhages, and necrosis, usually involving watershed areas of the splenic flexure and sigmoid.

Histologically, CC and IC are distinct entities. CC is characterized by thickening of the subepithelial collagen band, often associated with separation of the epithelium from the lamina propria, and lymphocytic infiltrates in the mucosa. IC at biopsy shows atrophic glandular crypts, hyalinizing fibrosis and often hemorrhage in the lamina propria with acute inflammation, micro-thrombi in the superficial capillaries, and, when severe, necrosis. Inflammatory pseudomembranes similar to those found in Clostridium difficile-associated pseudomembranous colitis may be present.

In the present case, the histologic findings typical of collagenous colitis were clearly present. However, as distinction from ischemic colitis is critical, we further examined the histologic sections with a view to eliminating concomitant ischemic colitis contributing to the clinico-pathological picture. A careful examination of the non-ulcerated mucosa showed no evidence of atrophic glandular crypts, hyalinizing fibrosis or hemorrhage in the lamina propria, or intra-capillary micro-thrombi. The absence of these changes allowed for confident exclusion of associated ischemic colitis. In contrast, photomicrographs in five of the six previously reported cases show the characteristic features of CC, but the authors did not explicitly address whether they considered ischemic colitis as a complicating factor (nor do the photomicrographs provided allow for exclusion of this complication). We believe it is worth keeping in mind that CC and IC should not be considered as mutually exclusive diagnoses.

Whereas risk factors, as described above, have been identified for iatrogenic perforation in CC, an explanation for spontaneous perforation is not so evident. One factor may be the degree of thickening of the collagen band, which in our case was focally severe, resulting in very tenuous integrity of the overlying epithelium. In this situation normal peristaltic activity or the repeated bowel movements characteristic of CC may have been sufficient to provide enough stress on the mucosa to lead to tearing and ulceration.

We conclude that, although decidedly rare, knowledge of spontaneous perforation as a possible complication of CC may be helpful in the evaluation and management of patients presenting with colonic perforation, especially those with risk factors for CC.

## Abbreviations

CC: Collagenous colitis
F: Female
